# Doxorubicin Differentially Induces Apoptosis, Expression of Mitochondrial Apoptosis-Related Genes, and Mitochondrial Potential in BCR-ABL1-Expressing Cells Sensitive and Resistant to Imatinib

**DOI:** 10.1155/2015/673512

**Published:** 2015-11-04

**Authors:** Ewelina Synowiec, Grazyna Hoser, Jolanta Bialkowska-Warzecha, Elzbieta Pawlowska, Tomasz Skorski, Janusz Blasiak

**Affiliations:** ^1^Department of Molecular Genetics, University of Lodz, Pomorska 141/143, 90-236 Lodz, Poland; ^2^Department of Flow Cytometry, Medical Center for Postgraduate Education, Marymoncka 99, 01-813 Warsaw, Poland; ^3^Department of Infectious and Liver Diseases, Medical University of Lodz, Kniaziewicza 1/5, 92-347 Lodz, Poland; ^4^Department of Orthodontics, Medical University of Lodz, Pomorska 251, 92-216 Lodz, Poland; ^5^Department of Microbiology and Immunology, School of Medicine, Temple University, Philadelphia, PA 19140, USA

## Abstract

Imatinib resistance is an emerging problem in the therapy of chronic myeloid leukemia (CML). Because imatinib induces apoptosis, which may be coupled with mitochondria and DNA damage is a prototype apoptosis-inducing factor, we hypothesized that imatinib-sensitive and -resistant CML cells might differentially express apoptosis-related mitochondrially encoded genes in response to genotoxic stress. We investigated the effect of doxorubicin (DOX), a DNA-damaging anticancer drug, on apoptosis and the expression of the mitochondrial NADH dehydrogenase 3 (*MT-ND3*) and cytochrome *b* (*MT-CYB*) in model CML cells showing imatinib resistance caused by Y253H mutation in the *BCR-ABL1* gene (253) or culturing imatinib-sensitive (S) cells in increasing concentrations of imatinib (AR). The imatinib-resistant 253 cells displayed higher sensitivity to apoptosis induced by 1 *μ*M DOX and this was confirmed by an increased activity of executioner caspases 3 and 7 in those cells. Native mitochondrial potential was lower in imatinib-resistant cells than in their sensitive counterparts and DOX lowered it. MT-CYB mRNA expression in 253 cells was lower than that in S cells and 0.1 *μ*M DOX kept this relationship. In conclusion, imatinib resistance may be associated with altered mitochondrial response to genotoxic stress, which may be further exploited in CML therapy in patients with imatinib resistance.

## 1. Introduction

Chronic myeloid leukemia (CML) is a clonal disease originating from aberrations in hematopoietic stem cell occurring on average rate as 1-2 cases per 100 000 [1]. This disease has three consecutive phases: the chronic (stable) phase, which may not produce any sign of disease, accelerated phase, and blast crisis, which is fatal. The occurrence of CML is associated with reciprocal chromosomal translocation between chromosomes 9 and 22, t(9;22)(q34;q11), resulting in formation of a fusion chromosome, the Philadelphia (Ph) chromosome [2]. This chromosome contains a chimeric gene, the* BCR-ABL1* gene, a fusion of the* ABL1* gene of chromosome 9 and a fragment of the* BCR* gene of chromosome 22. Depending on the point of breakage in* BCR*, the transcription of* BCR-ABL1* may result in three different RNAs, resulting in three isoforms of the BCR-ABL1 protein. CML patients have mostly the p210 isoform of BCR-ABL1. This protein has a constitutively increased tyrosine kinase activity and shows oncogenic properties, which are resulting from its ability to phosphorylate proteins involved in many cancer-related signaling pathways. It affects many properties of hematopoietic cells, giving them growth and proliferation advantage over normal counterparts and inducing resistance to apoptosis [3].

Several modes of therapy, including interferon-*α*, hydroxyurea, cytosine arabinoside, and allogenic stem cell transplantation, were applied in CML treatment, but introduction of imatinib mesylate (imatinib, Gleevec, STI 571) and other tyrosine kinase inhibitors significantly improved its efficacy [4]. However, cessation of taking imatinib may result in disease relapse, because a small population of leukemic cells can survive imatinib treatment and retain capacity to self-renew [5]. Another important problem related to imatinib use is emerging resistance to it, which can be primary or secondary. The former is usually associated with mutations in the* BCR-ABL1* gene, whereas the latter is acquired in the course of therapy. In both kinds of resistance, there may be a difference between the activities of certain proteins in resistant and sensitive cells [6]. This difference may result from the difference in the expression of genes encoding those proteins. Because the main mode of the action of imatinib is induction of apoptosis in CML cells, genes which are involved in apoptotic signaling may play an important role in differential sensitivity to imatinib [7]. Furthermore, apoptosis in a single cell is turned on after reaching some threshold just once and there is need to keep the threshold value continuously [8]. This threshold achievement occurs when antiapoptotic proteins of the Bcl-2 family (such as Bcl-2, Bcl-XL, or Mcl-1) are prevented from binding to the proapoptotic Bcl-2 family members Bax or Bak, which are then able to form pores in the outer mitochondrial membrane, which result in the cell death [9]. Therefore, some perturbations in the functioning of mitochondria may result in altered response of BCR-ABL1+ cells to imatinib. This also concerns mitochondrial DNA (mtDNA), as it may accumulate DNA damage due to high level of reactive oxygen species (ROS) in its environs and less efficient DNA repair in comparison with nuclear DNA [10]. Changes in mtDNA may result in alterations in the expression of mitochondrially encoded genes, which in turn may influence mitochondrial functions, disturbing the internal apoptotic pathway. Therefore, we hypothesize that imatinib resistance might be associated with the expression of mitochondrial genes. All 13 mitochondrial protein-encoding genes code for components of oxidative phosphorylation system (OXPHOS). Some of them may be associated with apoptotic or antiapoptotic signaling, which justifies their potential involvement in resistance against apoptosis-inducing drugs, including imatinib. The mitochondrial NADH dehydrogenase 3 (*MT-ND3*) gene codes for one of the mitochondrially encoded subunits of NADH dehydrogenase (ND3), which is composed of over 40 subunits—7 coded by mitochondrial and the rest coded by nuclear DNA [11]. It was shown that downregulation of MT-ND3 expression in a cancer cell line resulted in resistance to apoptosis induced by doxorubicin (DOX), an anticancer drug stabilizing DNA topoisomerase II cleavage complex and inducing reactive oxygen species (ROS) [12]. The mitochondrial cytochrome b (*MT-CYB*) gene coding for the MT-CYB protein (cytochrome b) is essential for the assembly and functioning of the complex III of OXPHOS. An overexpression of MT-CYB was reported to be involved in antiapoptotic signaling [13].

To verify the hypothesis that the expression of mitochondrial genes may be associated with imatinib resistance we evaluated mRNA levels of the* MT-ND3* and* MT-CYB* genes expression in imatinib-sensitive and imatinib-resistant model leukemic cell lines. We used BCR-ABL1-expressing lines with both primary and acquired imatinib resistance. In addition, because DNA damage is archetypal factor influencing apoptotic pathways, we checked MT-ND3 and MT-CYT expression and the extent of apoptosis upon DOX treatment. Doxorubicin induces double strand DNA breaks in proliferating cells due to inhibition of DNA topoisomerase II and much other DNA damage induced by ROS produced by this drug in both cancer and normal cells [14]. Protection from apoptosis is a major BCR-ABL1-induced carcinogenic effect and its mechanism is not completely known; many pathways may be involved, including inhibition of mitochondrial cytochrome c release and inhibition of caspase activation [15]. Mitochondrial dysfunctions may be crucial for the apoptotic pathway and can be related to depolarization of the mitochondrial membrane potential (MMP) [16]. Therefore, it seems justified to consider MMP as a factor which might also play a role in imatinib resistance.

## 2. Materials and Methods

### 2.1. Chemicals

Imatinib was a kind gift of Novartis (Basel, Switzerland). IMDM was purchased from Gibco BRL (Basel, Switzerland). MTT and doxorubicin (DOX) were obtained from Sigma-Aldrich (St. Louis, MO, USA). AxyPrep Multisource Total RNA Miniprep Kit was purchased from Axygen Biosciences, Union City, CA, USA. High Capacity cDNA Reverse Transcription Kit and probes for genes expression with TaqMan Gene Expression Assay were obtained from Life Technologies (Grand Island, NY, USA). Apoptosis kit was purchased from BD Biosciences (San Jose, CA, USA). MitoProbe JC-1 Assay Kit was purchased from Life Technologies.

### 2.2. Cells and Treatment

Murine 32D clone 3 cell line transfected with p210 BCR-ABL1 was obtained from Dr. T. Skorski of Temple University (Philadelphia, PA, USA). The parental cells were transfected with a native BCR-ABL1 or its mutated variant, carrying the Y253H mutation, conferring the resistance to imatinib in these cells. A part of cells with native BCR-ABL1 was cultivated in a growing imatinib concentration, acquiring resistance to this drug. This was reached by incubating the sensitive cells with 0.01 *μ*M imatinib with the replacement of the medium every day. After 2-3 weeks, when survival rate reached 80%, incubation was continued with imatinib concentration 0.05 *μ*M and this procedure was repeated with growing concentrations of imatinib until at least 80% of cells survived at 1 mM imatinib. Therefore, we used three 32D cell lines with BCR-ABL1 expression: sensitive to imatinib, referred to as S, and a primarily imatinib-resistant line Y253H (253) as well as the line with acquired imatinib resistance (AR). The cells were grown in IMDM, a modified Dulbecco's medium supplemented with 2 mM L-glutamine, 100 U/mL penicillin, 100 *μ*g/mL streptomycin, and 10% fetal bovine serum and maintained at 37°C in 5% CO_2_ atmosphere at 100% humidity.

Where indicated, the cells were incubated at 37°C with 1.0 *µ*M DOX for 24 h, and then the drug was washed out and the cells were further analyzed.

### 2.3. MTT Assay for Cell Viability

To determine cell viability after imatinib treatment the MTT assay was used. 32D cells (5 × 10^4^/100 *µ*L per well) were cultured in a 96-well plate at 37°C and exposed to varying concentrations of imatinib (0.01–1 *µ*M) for 24 h. Cells treated with culture medium alone served as negative control. Subsequently, cells were washed once with PBS and incubated with 0.5 mg/mL of thiazolyl blue tetrazolium bromide (MTT) solution in PBS at 37°C for 4 h. Following incubation, the MTT was discarded carefully and 100 *µ*L dimethyl sulfoxide (DMSO) was added to solubilize the formazan crystals. Finally, the absorbance was measured for each well at 595 nm with a reference wavelength of 655 nm, using Microplate Reader 550 (Bio-Rad Laboratories, Hercules, CA, USA). Each measurement was repeated six times. Cell viability was expressed as a fraction of treated to untreated living cells.

### 2.4. Gene Expression

Total RNA was extracted from 5 × 10^6^ cells using ISOLATE II RNA Mini Kit (Bioline Reagents Ltd., London, UK) and stored in TE buffer (5 mM Tris-HCl, 0.1 mM EDTA, and pH 8.5 in DEPC-treated water) at −20°C until further analysis. First-strand cDNA was synthesized from total RNA using High Capacity cDNA Reverse Transcription Kit. A sample of 2 ng of total RNA was used as a template in a total volume of 20 *µ*L. Real-time quantitative PCR for murine NADH dehydrogenase subunit 3 (ND3, assay ID: Mm04225292_g1), murine cytochrome b (CYB, assay ID: Mm04225271_g1), and murine *β*-actin (Actb, assay ID: Mm00607939_s1) was performed using the TaqMan Gene Expression Assay in a thermal cycler CFX96 Real-Time PCR Detection System (BIO-RAD Laboratories, Hercules, CA, USA). The thermal cycling conditions were as follows: 10 min of polymerase activation at 95°C, followed by 40 cycles of 30 s denaturation at 95°C and 60 s annealing/extension at 60°C. Each sample was run in duplicate. The cycle threshold (Ct) values were calculated by CFX96 Real-Time PCR Detection System (BIO-RAD) software and the comparative Ct method (2^−ΔCt^ model) was used to calculate relative fold-changes in gene expression and normalized to the average of Actb.

### 2.5. Apoptosis

Apoptosis was evaluated by flow cytometry with Dead Cell Apoptosis Kit with annexin V APC and SYTOX Green (Molecular Probes). The cells were washed twice in PBS and suspended in 100 *µ*L 1x annexin-binding buffer at the cells' density of 10^6^. A 5 *μ*L APC annexin V and 1 *μ*L of the 1 *μ*M SYTOX Green were added to each 100 *μ*L cell suspension, which was then incubated at 37°C in an atmosphere of 5% CO_2_ for 15 min. After the incubation period, 400 *μ*L of the 1x annexin-binding buffer was added and samples were analyzed by flow cytometry on LSRII (Becton Dickinson, San Jose, CA, USA) flow cytometer and 5 × 10^5^ cells were analyzed per sample in three independent experiments. Quadrants were set on the basis of control samples exposed to camptothecin (4 *µ*M, 12 h incubation). Apoptotic index was calculated as the mean percentage of apoptotic cells in 5 × 10^4^ cells measured in each of three independent experiments.

### 2.6. Caspases Activity

To determine the activity of caspase-3/caspase-7, CellEvent Caspase-3/7 Green Detection Reagent (Molecular Probes, Invitrogen, Carlsbad, CA) was used. This reagent is a fluorogenic substrate for activated caspases 3 and 7 and consists of four-amino acid peptide (DEVD) conjugated to a nucleic acid binding dye (nonfluorescent in live cells). In apoptotic cells, after activation of caspase-3/caspase-7, the DEVD is cleaved, enabling the dye to bind to DNA and produce a bright, fluorogenic response with absorption/emission maxima of ~502/530 nm. Briefly, 5 × 10^4^ cells per well were seeded in black 96-well plates and cultured overnight. The next day, doxorubicin (DOX) was added to the cells. After 24 h of incubation, CellEvent Caspase-3/7 Green Detection Reagent was added to the wells at a final concentration of 5 *µ*M. The caspase activity was determined after 30 min of incubation by measuring the fluorescence intensity of cells, at 502 nm excitation wavelength and an emission wavelength of 530 nm, using a Bio-Tek Synergy HT Microplate Reader. Each sample was run in quadruplicate.

### 2.7. Mitochondrial Membrane Potential

MMP was determined by MitoProbe JC-1 Assay Kit (Life Technologies) using fluorescence microplate readers and fluorescence microscopy. The kit contains the cationic dye JC-1 (5′,6,6′-tetrachloro-1,1′,3,3′-tetraethylbenzimidazolylcarbocyanine iodide) and a mitochondrial membrane potential disrupter CCCP (carbonyl cyanide 3-chlorophenylhydrazone). This carbocyanine dye accumulates in the mitochondrial membrane in a potential-dependent manner. High potential of the inner mitochondrial membrane facilitates formation of the dye aggregates (J-aggregates) with both excitation and emission shifted towards red light (530 nm/590 nm) when compared with that for JC-1 monomers (485 nm/538 nm). Cells were seeded into 96-well black plate at a density of 5 × 10^4^ cells/well in 100 *µ*L culture medium, incubated with or without experimental compounds and cultured in CO_2_ incubator at 37°C for 24 h. Each experiment included a positive control; 10 *μ*M of the CCCP was added to the cells as an uncoupler of mitochondrial oxidation. Finally, the cells were preincubated with 5 *μ*M JC-1 in the HBSS in CO_2_ incubator at 37°C for 30 min. Before measurements, the cells were centrifuged (300 g for 10 min at 22°C) and then washed twice with the HBSS. The fluorescence was measured on Bio-Tek Synergy HT Microplate Reader (Bio-Tek Instruments, Winooski, VT, USA) with the filter pairs of 530 nm/590 nm and 485 nm/538 nm. Results are shown as a ratio of fluorescence measured at 530 nm/590 nm to that measured at 485 nm/538 nm (aggregates to monomer fluorescence). Each sample was run in quadruplicate.

For fluorescence microscopy, cells were cultured on a 6-well plate at a density of 1 × 10^6^ cells/well in a 5 mL culture medium, treated with DOX (1 *µ*M) and placed in a CO_2_ incubator at 37°C for 24 h. At the end of the incubation, cells were stained with 10 *µ*M JC-1 in HBSS and once more placed in 37°C for 30 min. Next, the cells were centrifuged (300 g for 10 min at 22°C) and then washed twice and finally mounted in HBSS on microscope slides and visualized using Olympus inverted fluorescence microscope (Olympus IX70, Japan).

### 2.8. Data Analysis

Six replications were analyzed for each cell line in cell viability assay and remaining experiments were repeated 3-4 times. The differences between samples were evaluated by Student's test. Measurements with real-time PCR were performed in triplicate and one-way ANOVA was used with Holm-Sidak multiple comparison method. All statistical analyses were performed with GraphPad Prism v. 5.00 for Windows (GraphPad Software, San Diego, CA, USA).

## 3. Results

### 3.1. Cell Viability

**Figure 1:**
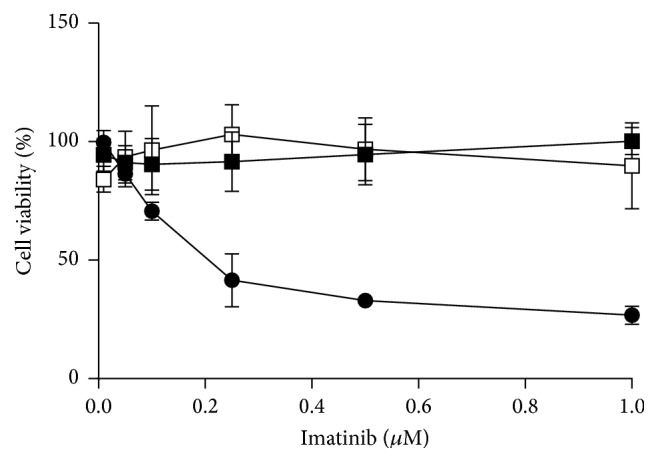
Cell viability of mouse-derived 32D cells transfected with the* BCR-ABL1* oncogene sensitive to imatinib (filled circles) or cells with primary resistance to imatinib caused by the Y253H mutation in* BCR-ABL1* (filed squares) or acquired imatinib resistance (empty squares). Viability was evaluated by MTT assay. Each point is mean of six independent experiments; error bars represent SEM but in some points they were smaller than the symbol radius.

Imatinib evoked a significant (*p* < 0.005) decrease in the viability of imatinib-sensitive cells as compared with unexposed cells and both primary and secondary imatinib-resistant cells (Figure 1). The parental cells, nontransfected with BCR-ABL1, were also resistant to imatinib up to its 1 *µ*M concentration. Although imatinib is reported to inhibit the ABL kinase in normal cells, they have other kinase activities, which are not affected by imatinib [17]. The decrease in the viability in sensitive cells exceeded 80% at 1 *µ*M imatinib. Therefore, we considered the S subline to be sensitive to imatinib as compared to remaining ones, considered to be resistant. The values of viability exceeding 100% observed for the P line might result from an excessive proliferation of this line in the presence of interleukin-3 or several other reasons, including direct interaction of imatinib with tetrazolium (MTT) dye or stimulating effect of imatinib on mitochondrial enzymes reducing MTT to formazan. However, although the MTT assay has several limitations [18], we found it suitable for our study as it clearly shows systematic differences in the viability between sublines.

### 3.2. Mitochondrial Cytochrome b and NADH Dehydrogenase 3 Basal Expression

**Figure 2:**
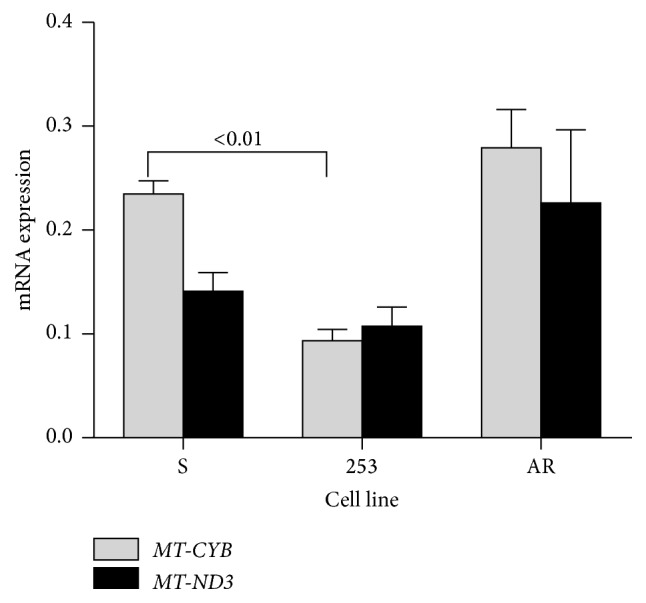
Basal mRNA expression of mitochondrial cytochrome b (*MT-CYB*) and NADH dehydrogenase 3 (*MT-ND3*) genes determined by real-time PCR in mouse-derived 32D BCR-ABL1+ cells sensitive to imatinib (S) and cells with resistance caused by the Y253H mutation in the* BCR-ABL1* gene (253) or acquired imatinib resistance (AR). The expression of either gene was normalized to the mouse*β-actin* gene;* n* = 3 for each cell line; number above bars shows significant* p* values for comparison between pairs connected by brackets.

We observed a significant (*p* < 0.01) decrease in mRNA MT-CYB expression in 253 imatinib-resistant cells as compared with the sensitive line (Figure 2). There were no differences between mRNA expression of either gene in AR and sensitive cells. Therefore, these results suggest different mechanisms of regulation of* MT-CYB* and* MT-ND3* expression in imatinib-resistant 253 and AR cells.

### 3.3. MT-CYB and MT-ND3 Expression after Doxorubicin Treatment

**Figure 3:**
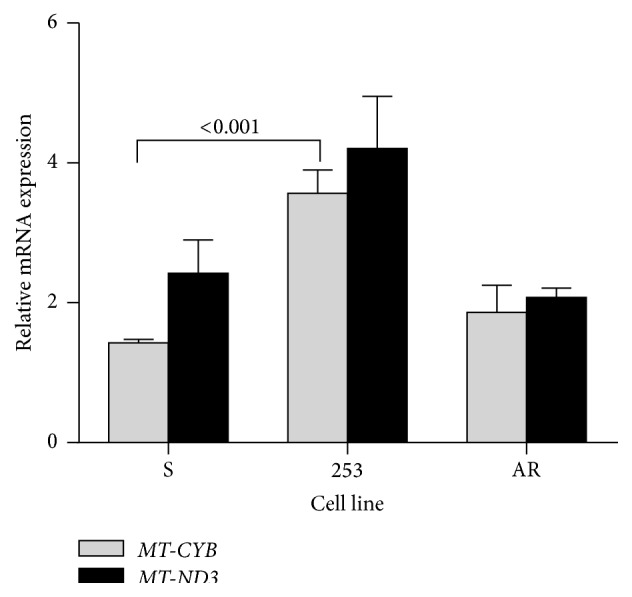
Relative mRNA expression of mitochondrial cytochrome b (*MT-CYB*) and NADH dehydrogenase 3 (*MT-ND3*) genes determined by real-time PCR in mouse-derived 32D BCR-ABL1+ cells sensitive to imatinib (S) or cells with imatinib resistance caused by the Y253H mutation in the* BCR-ABL1* gene (253) or acquired imatinib resistance (AR). The cells were exposed at 37°C for 24 h to doxorubicin (DOX) at 1.0 *µ*M. Presented is a ratio of expression for DOX-exposed and DOX-nonexposed cells. The expression of either gene was normalized to the mouse*β-actin* gene;* n* = 3 for each cell line; the numbers above bars indicate significant* p* values for comparison between pairs connected by brackets.

Doxorubicin at 1.0 *µ*M evoked increase in the mRNA expression of both* MT-CYB* and* MT-ND3* genes in all kinds of BCR-ABL1-transfected cells (Figure 3). The relative mRNA expression of the* MT-CYB* gene in the 253 imatinib-resistant cells was significantly higher than that in their imatinib-sensitive counterparts. Therefore, the Y253H mutation in the* BCR/ABL1* gene influences expression of* MT-CYB* in the presence of DOX.

### 3.4. Apoptosis after Doxorubicin Treatment

**Figure 4:**
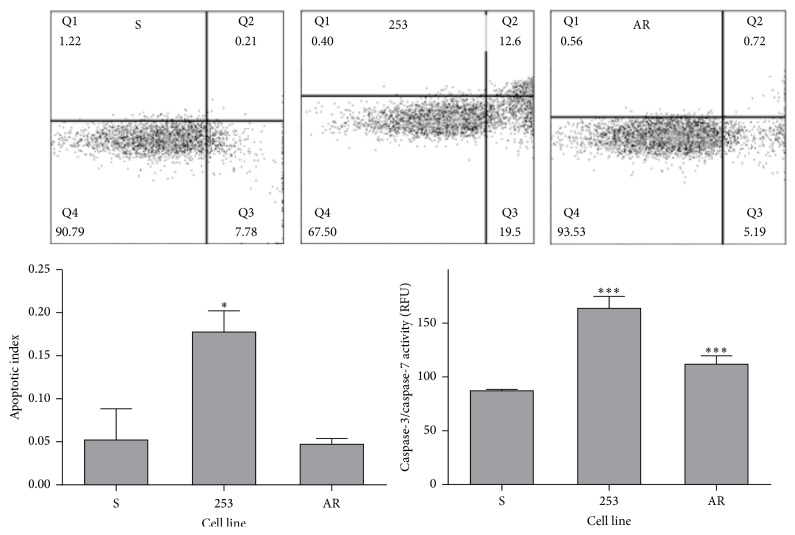
Apoptosis in mouse-derived 32D BCR-ABL1+ cells sensitive to imatinib (S) and cells with imatinib resistance caused by the Y253H mutation in the* BCR-ABL1* gene (253) or acquired imatinib resistance (AR). The cells were exposed at 37°C for 24 h to doxorubicin (DOX) at 1 *µ*M. Apoptosis was evaluated by flow cytometry with annexin V APC and SYTOX Green dyes. Apoptotic index was calculated as the percentage of apoptotic cells in 5 × 10^4^ cells measured in each of three independent experiments. The dot plots show results of one representative experiment for each kind of cells treated with DOX. The number in the corner of Q3 quadrant presents a fraction of early and late apoptotic cells. The activity of caspases 3 and 7 was determined fluorometrically with CellEvent Caspase-3/7 Green Detection Reagent and presented in relative fluorescent units (RFU). ^*∗*^ *p* < 0.05 and ^*∗∗∗*^ *p* < 0.001 as compared with imatinib-sensitive cells.

Doxorubicin at 1.0 *µ*M induced apoptosis in all kinds of cells (Figure 4). However, apoptotic index in imatinib-resistant cells with Y253H mutation was significantly higher than that in the remaining cell lines. The activity of caspases 3 and 7 confirmed this relationship, although the increase of activity for the 253 line in comparison with S cells was not statistically significant. This is in line with results obtained in experiment with the expression of mitochondrial apoptosis-related genes, but the process of apoptosis results from a complex, multiprotein signaling, which can be changed in imatinib-resistant cells bearing the Y253H mutation.

### 3.5. Mitochondrial Membrane Potential

**Figure 5:**
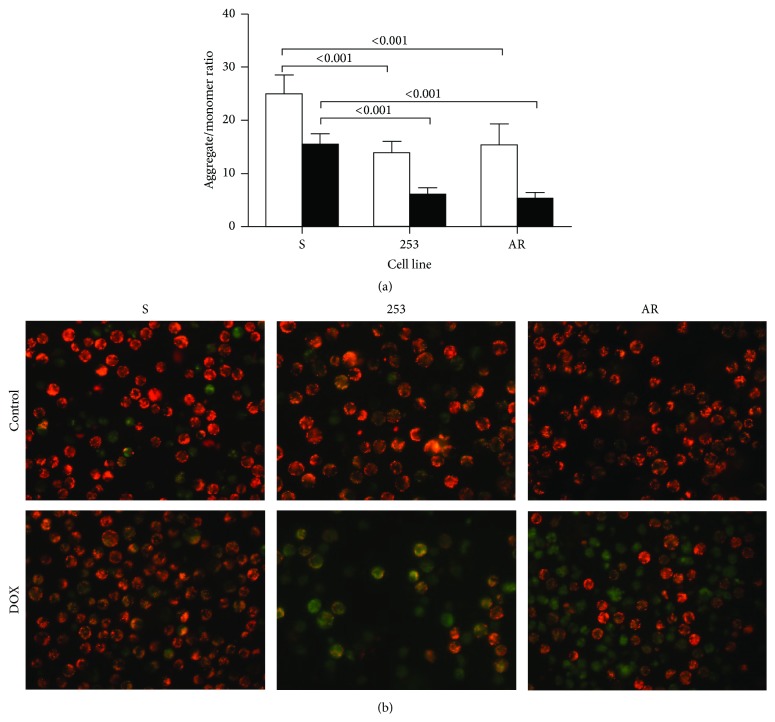
Mitochondrial membrane potential expressed as JC-1 aggregate to monomer ratio in mouse-derived 32D BCR-ABL1+ cells sensitive to imatinib (S) and cells with imatinib resistance resulted from the Y253H mutation in the* BCR-ABL1* gene (253) or acquired imatinib resistance (AR) (a). The cells were exposed at 37°C for 24 h to doxorubicin (DOX) at 1 *µ*M (black bars) or unexposed (control, empty bars). Each experiment was repeated four times and error bars denote SD. Numbers indicate significant* p* values for comparison between pairs connected by brackets. Fluorescent microscopy images of control (untreated) cells and cells treated with 1 *µ*M DOX (b). Red fluorescence of JC-1 dimers is present in the cell areas with high mitochondrial membrane potential, while green fluorescence of JC-monomers is prevalent in the cell areas with low mitochondrial membrane potential. The JC-1 stained cells were visualized under an Olympus inverted fluorescence microscope, model IX70 (Olympus, Tokyo, Japan) with 400x magnification.

We observed a significant lowering of aggregate/monomer ratio in imatinib-resistant cells as compared with their sensitive counterparts (Figure 5). Doxorubicin at 1.0 *µ*M decreased MMP in all kinds of cells and imatinib-resistant cells had MMP still significantly lower than their sensitive counterparts.

## 4. Discussion

Genomic instability is a hallmark of cancer cells and it may determine their reaction to genotoxic stress. The BCR-ABL1 oncogene induces genomic instability in CML cells and its effect may be different on imatinib-sensitive and -resistant cells [15]. Therefore, the reaction to genotoxic stress may be different in imatinib-resistant and -sensitive cells and this difference may result from the interaction of BCR-ABL1 with its signaling partners. The identification of these partners might contribute to CML therapy in imatinib-resistant patients. In this work we employed doxorubicin to induce genotoxic stress and searched for the association between the expression of mitochondrial MT-CYB and MT-ND3 genes and imatinib resistance. We chose these genes because their expression may be associated with apoptosis and imatinib is apoptosis-inducing drug. Mitochondria may be involved in imatinib resistance as they play an essential role in the intrinsic apoptotic pathway. Moreover, it was shown that imatinib-resistant, BCR-ABL1-expressing cells, K562 and LAMA84, had a highly glycolytic metabolic phenotype characterized by a high glucose use and lactate production [19]. However, increased glucose metabolism in BCR-ABL1 positive cells is suggested to associate with increased mitochondrial production of ROS due to overactive mitochondrial electron transport [20].

Our research was performed on single, murine-derived 32D cell line, which is an apparent limitation of our study. However, we created a fairly unique CML model system, representing CML in its chronic phase with imatinib sensitivity (S cells) and CML with various determinants of imatinib resistance. Creating such a system using primary human CML cells requires long time and enrolling many CML patients. We see our study as an introduction to the field of the role of mitochondrial metabolism in imatinib resistance. In perspective, LAMA84 human cell line, corresponding to CML blast crisis, can be used, but mechanism of imatinib resistance is largely not known in these cells [21]. In turn, K562 human CML cells resistant to imatinib show multidrug resistance, which is BCR-ABL1 independent [22].

To obtain our experimental CML model, we transfected mouse bone marrow cells with a retroviral virus and then tested this model by studying some mitochondrial apoptosis-related parameters in these cells. Therefore, it is important to consider whether the process of transfection might affect these parameters. Retroviral transfection is a recombinant event with DNA double strand breaks, as an intermediate. These breaks, if not resealed, can induce mitochondrial dysfunctions and in this way affect mitochondrial apoptosis [23].

We related our results to values obtained for parental cells, which are not good control as they do not have the* BCR-ABL1* gene. Although they were transfected by an empty vector, they have a different genome so they are not suitable control for all cell lines. Moreover, including the net results for the parental 32D cells would provoke a discussion on the effect of BCR-ABL1 on apoptosis-related phenomena, which is not a subject of this work. Lastly, parental cells have nothing to do with imatinib. That is why we considered imatinib-sensitive cells as the control and values we obtained for parental cells were used as the units of quantities we measured.

We observed that 1.0 *µ*M DOX evoked apoptosis in all cell lines, but apoptotic index, indicating the fraction of cells undergoing apoptosis, was significantly lower in S cells than in the remaining cell lines (Figure 4). The observation that the S subline was more resistant to apoptosis than the parental P lines is in agreement with the basic feature of BCR-ABL1 to protect against apoptosis induced by genotoxic treatment. However, the 253 and AR lines also express BCR-ABL1, but we did not observe any antiapoptotic effect on these lines. It was shown that the Y253H mutation induced a growth advantage only in the presence of imatinib [24]. The impact of the Y253H mutation on the general oncogenic properties of BCR-ABL1 is largely unknown and, moreover, some divergent results were reported.

The Y253H mutation occurs at the nucleotide-binding loop domain of the active center of BCR-ABL1, changing its conformation and preventing imatinib binding [25]. This effect may be caused by impairing of the induced-fit interaction of imatinib with the active center compared to sterically blocking its binding. It is worth noting that CML patients with a P-loop mutation were reported to have particularly poor prognosis [26]. It was concluded that the Y253H mutation had not a strong influence on the transforming properties of BCR-ABL1. Therefore, we cannot a priori assume that the 253 cells should behave similarly to S cells in expressing apoptosis in response to genotoxic treatment. The interpretation of apoptotic behavior of AR cells after DOX action is even more complex due to unknown nature to imatinib resistance in these cells. Many effects may be involved in such kind of imatinib resistance, including alternative splicing, inducing noncanonical BCR-ABL1 signaling pathways and epigenetic changes [27]. Therefore, our data suggest that imatinib resistance, both primary and secondary, may not be associated with general apoptotic signaling in the absence of imatinib.

Our results show that different mechanisms are involved in imatinib resistance in 253 and AR cells. Moreover, our experience with culturing AR cells indicates that they are dynamic entities and mechanisms involved in their imatinib resistance can change in time. Due to unknown nature of genetic and epigenetic alterations in the AR cells, we cannot assess possible clonal expansion of a subpopulation of these cells, which might determine their imatinib resistance.

We speculate that imatinib resistance in AR cells may, at least in part, result from mutation(s) in the* BCR/ABL1* gene. Moreover, we detected the Y253H mutation, but not T315I, in a fraction of AR culture (data not shown). Therefore, imatinib resistance in AR cells can be caused by complex and dynamic mechanisms, including mutation in the* BCR/ABL1* gene. This is in general in line with the picture of acquired imatinib resistance in CML patients [26].

We used two different DOX concentrations—0.1 and 1.0 *µ*M. The former is typically used in experiments with DOX-induced DNA damage, whereas the latter is usually employed to induce apoptosis. Doxorubicin can induce a broad spectrum of DNA damage [27]. Its primary anticancer action is based on stabilization of DNA topoisomerase II-DNA cleavage complex, which leads to the inhibition of cancer cells replication and transcription and DNA fragmentation [28]. However, DOX, possibly thanks to its structure, also stimulates ROS production, which may induce various DNA damage, first of all oxidative base modifications, especially at moderate DOX concentrations [29]. If endogenous formaldehyde is available at the site of DOX action, the drug may form DOX-DNA adducts, which may constitute a serious challenge for DNA repair pathways, as they can act as interstrand cross-links [27, 29]. Because DNA damage is archetype of cell cycle arresting and apoptosis-inducing factor, we observed* MT-CYB* and* MT-ND3* expression on the initiating stages of apoptotic pathway and not in its advanced stages, when changes induced by apoptosis itself may play an important role in the expression of these genes.

It should be taken into account that doxorubicin may inhibit the nuclear fluorescence of cells stained with propidium iodide [29]. Therefore, as PI-positive cells are typically in late apoptosis or already dead, apoptotic index we calculated may be slightly underestimated. Mitochondrial membrane potential plays an important role in mitochondrial apoptotic pathway and its loss is associated with apoptosis [2]. Changes in MMP may be linked with formation of pores in mitochondrial outer membrane and leakage of cytochrome c [30]. As showed by Petronilli et al. [31] mitochondria of imatinib-resistant cells displayed a variety of mitochondrial dysfunctions, at least in part associated with ROS-overproduction in such cells. They showed that imatinib-resistant cells displayed a slightly lower MMP than their imatinib-sensitive counterparts. Also, a lower oxygen intake rate in the resistant cells was observed, which was explained by a decrease in ATP turnover and decrease in the proton leak. An accumulation of tricarboxylic acid as well as decreased level of glutamate was also observed in that study. This, along with simultaneous increase in NADH concentration, suggests deregulation of mitochondrial respiratory chain (MRC) in imatinib-resistant cells, which is in agreement with our previous work showing a high level of ROS accumulation in imatinib-resistant cells [32]. Imatinib was reported to disrupt MMP in melanoma cells and this effect was associated with ROS-dependent apoptosis induced by imatinib [33]. Imatinib reduced MMP in BCR-ABL1-expressing cells with concurrent Bax activation, but this activation occurred only when imatinib was in combination with nutlin-3, an inhibitor of Mdm2, a key regulator of p53 [33, 34]. Therefore, imatinib-dependent disruption of MMP may be associated with apoptotic events and proteins involved in apoptosis. Our results confirm and extend this knowledge on the expression of the mitochondrial* MT-CYB* and* MT-ND3* genes, whose products may be involved in apoptosis. However, our study was performed on mRNA, so it should be prospectively supplemented with research on functionality of the protein products of these genes to assure the association of expression, mitochondrial potential, and apoptosis with imatinib resistance.

The results obtained in this study are in general agreement with our previous work, in which we showed a different reaction of imatinib-resistant and -sensitive cells to genotoxic stress induced by UV radiation, which was indicated by different ROS production and DNA damage and apoptosis in these cells [35].

In conclusion, the results we obtained suggest that imatinib resistance may be generally associated with mitochondrial dysfunction and genotoxic treatment, inducing or increasing genomic instability, strengthens this association. Therefore, mitochondria in general and mtDNA in particular may be considered as a potential target in imatinib-resistant CML patients.

## 5. Conclusions

The results presented suggest that different effects of doxorubicin in imatinib-resistant and -sensitive cells may be associated with some mitochondrial dysfunctions, which may be linked to the inner apoptotic pathway. Therefore, mitochondria in general and especially mtDNA may be considered as a potential target in imatinib-resistant CML patients.
